# Supplementary material for: Novel Driver Strength Index highlights important cancer genes in TCGA PanCanAtlas patients
Source: PeerJ. 2022 Aug 11;10:e13860. doi: 10.7717/peerj.13860 (PMC9375969; doi:10.7717/peerj.13860)
Supplement: Supplemental Information 2 [file peerj-10-13860-s002.zip › COHORTS/genes plots/detailed/2021_8_16_14_9_distribution_events_detailed_8.pdf]

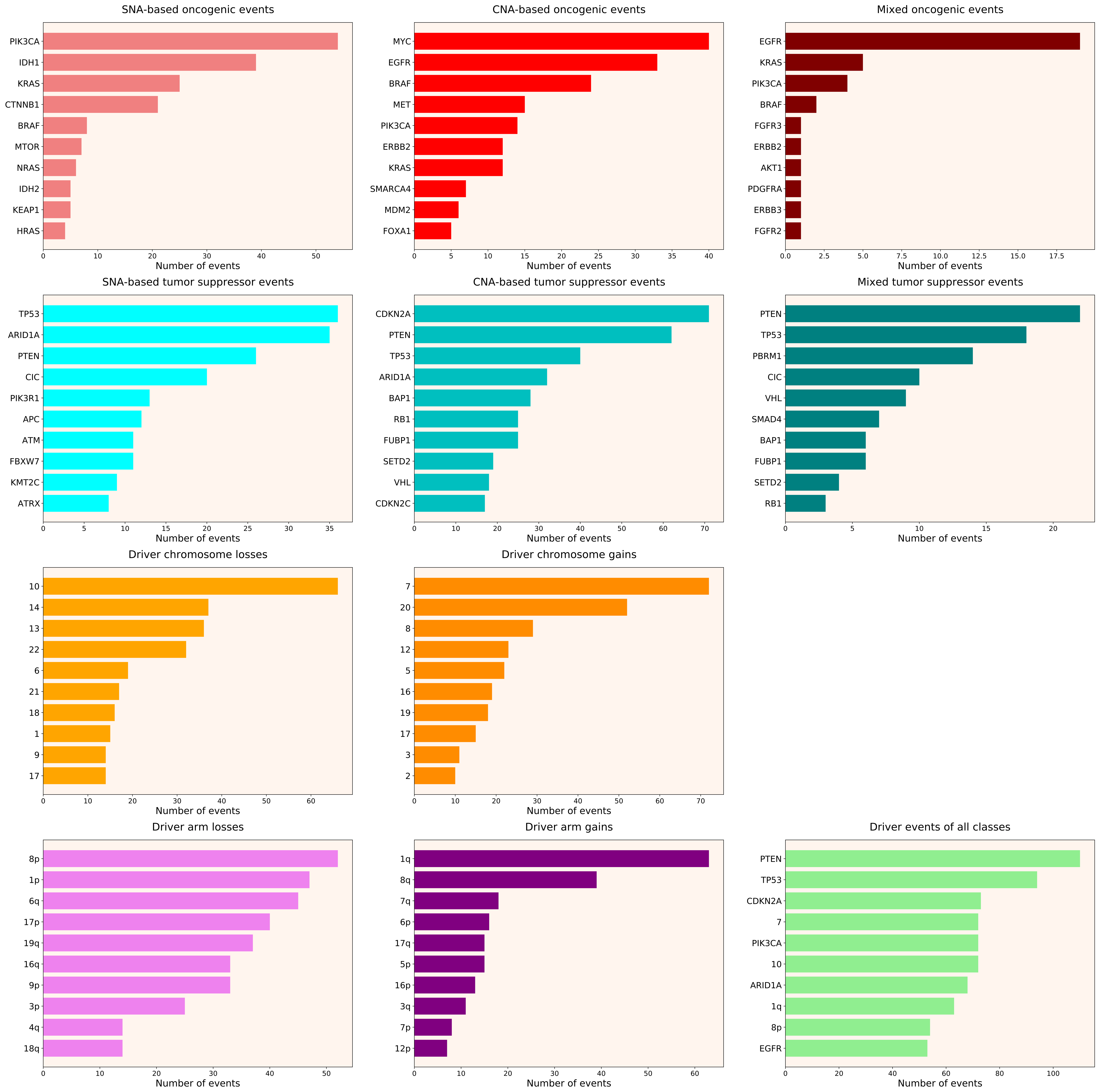
SNA-based oncogenic events
PIK3CA
IDH1
KRAS
CTNNB1
BRAF
MTOR
NRAS
IDH2
KEAP1
HRAS
0 10 20 30 40 50
Number of events
CNA-based oncogenic events
MYC
EGFR
BRAF
MET
PIK3CA
ERBB2
KRAS
SMARCA4
MDM2
FOXA1
0 5 10 15 20 25 30 35 40
Number of events
Mixed oncogenic events
EGFR
KRAS
PIK3CA
BRAF
FGFR3
ERBB2
AKT1
PDGFRA
ERBB3
FGFR2
0.0 2.5 5.0 7.5 10.0 12.5 15.0 17.5
Number of events
SNA-based tumor suppressor events
TP53
ARID1A
PTEN
CIC
PIK3R1
APC
ATM
FBXW7
KMT2C
ATRX
0 5 10 15 20 25 30 35
Number of events
CNA-based tumor suppressor events
CDKN2A
PTEN
TP53
ARID1A
BAP1
RB1
FUBP1
SETD2
VHL
CDKN2C
0 10 20 30 40 50 60 70
Number of events
Mixed tumor suppressor events
PTEN
TP53
PBRM1
CIC
VHL
SMAD4
BAP1
FUBP1
SETD2
RB1
0 5 10 15 20
Number of events
Driver chromosome losses
10
14
13
22
6
21
18
1
9
17
0 10 20 30 40 50 60
Number of events
Driver chromosome gains
7
20
8
12
5
16
19
17
3
2
0 10 20 30 40 50 60 70
Number of events
Driver arm losses
8p
1p
6q
17p
19q
16q
9p
3p
4q
18q
0 10 20 30 40 50
Number of events
Driver arm gains
1q
8q
7q
6p
17q
5p
16p
3q
7p
12p
0 10 20 30 40 50 60
Number of events
Driver events of all classes
PTEN
TP53
CDKN2A
7
PIK3CA
10
ARID1A
1q
8p
EGFR
0 20 40 60 80 100
Number of events
